# Neuropathic causes of groin pain in athletes: understanding nerve involvement

**DOI:** 10.1007/s00264-025-06461-z

**Published:** 2025-03-03

**Authors:** Zarko Vuckovic, Milos Bojovic

**Affiliations:** 1 VS Clinic, Belgrade, Serbia; 2 Aspetar, Orthopedic and Sports Medicine Hospital, Doha, Qatar

**Keywords:** Groin pain, Athletes, Inguinal-related groin pain, Nerve entrapment, Neuropathic pain, Nociceptive pain, Ilioinguinal nerve, Surgical treatment

## Abstract

**Purpose:**

Groin pain in athletes, particularly inguinal-related groin pain, remains a diagnostic and therapeutic challenge despite recent consensus on terminology. This study aims to explore nerve disorders as a key contributor to groin pain in athletes, focusing on the anatomy, aetiology, diagnosis, and management options.

**Methods:**

A comprehensive review of the literature was conducted, focusing on the anatomical variability of the ilioinguinal, iliohypogastric, and genital branch of the genitofemoral nerves, clinical presentations, diagnostic methods, and conservative and surgical treatments for nerve-related inguinal pain. Key studies on nerve entrapment, neuropathic and nociceptive pain mechanisms, and surgical outcomes were analyzed.

**Results:**

Variability in nerve pathways and sensory overlap complicate diagnosis and management. Neuropathic pain often presents with burning or electric sensations due to nerve compression or entrapment, while nociceptive pain manifests as dull or stabbing pain. Conservative treatment, including exercise-based rehabilitation and nerve blocks, offers relief in many cases. For refractory cases, surgical treatment can provide significant pain resolution, with nerve identification and potential neurectomy improving outcomes.

**Conclusion:**

Nerve disorders play a critical role in inguinal-related groin pain in athletes. Accurate diagnosis relies on detailed clinical examination and targeted imaging. Conservative treatments are first line, but surgical interventions addressing nerve entrapment or compression are effective for persistent cases. Future research should focus on the role of collagen deficiencies, nerve histopathology, and long-term outcomes of different treatment modalities.

## Introduction

Groin pain in athletes has been a perplexing medical challenge for decades, leading to uncertainty among healthcare professionals involved in diagnosing and treating athletes. Despite significant research efforts over the years, many questions remain unanswered. This condition predominantly affects athletes engaged in sports that require kicking, rapid changes in direction, and high-intensity movements [1]. A study investigating the prevalence of groin pain in football across different competition levels found that 59% of male players and 45% of female players experienced at least one episode of groin pain within a six-week period [2].

Beyond its substantial impact on athletic performance, groin pain presents a considerable challenge for sports medicine physicians, surgeons, and physiotherapists due to the complexity of its underlying pathology and the inconsistency in terminology and diagnostic definitions. To address this issue, a consensus on groin pain terminology and classification was reached during the First World Conference on Groin Pain in Athletes, held in Doha, Qatar, in 2014. The following year, a publication outlined the primary clinical entities associated with groin pain, categorizing them as adductor-related, iliopsoas-related, inguinal-related, and pubic-related, with hip-related groin pain recognized as a closely associated entity. Additionally, various medical conditions, including gastrointestinal, urogenital, and skeletal disorders, must be considered as potential sources of pain mimicking activity-related groin discomfort.

Inguinal-related groin pain is characterized by discomfort localized to the inguinal canal region, accompanied by tenderness in this area. Notably, there is no palpable inguinal hernia. The likelihood of this diagnosis increases if the pain is exacerbated by resistance testing of the abdominal muscles or during actions such as Valsalva maneuvers, coughing, or sneezing [3]. This article will specifically focus on inguinal-related groin pain, as there is ongoing debate among experts regarding its exact cause, and it remains one of the most challenging conditions to diagnose through clinical examination.

## Anatomy

The inguinal region represents the lower lateral section of the anterior abdominal wall, with the inguinal canal serving as its central anatomical feature. This area is where the lateral abdominal muscles connect with the pelvis, the rectus abdominis muscle, and the hip adductors. Additionally, the iliopsoas muscle is situated just below the inguinal ligament. The inguinal canal itself is composed of the external oblique aponeurosis anteriorly, the inguinal ligament inferiorly, the internal oblique and transversus abdominis muscles superiorly, and the transversalis fascia posteriorly. In males, the spermatic cord passes through the canal, while in females, the round ligament traverses the same structure, along with associated blood vessels and nerves.

This anatomical region plays a crucial role in sports movements, as it serves as a junction where forces from the abdominal wall and lower limbs converge. Consequently, the inguinal canal is considered one of the weak points of the abdominal wall, making it vulnerable to strain and injury.

Three primary sensory nerves traverse the inguinal canal: the ilioinguinal nerve, the iliohypogastric nerve, and the genital branch of the genitofemoral nerve. The ilioinguinal and iliohypogastric nerves originate from the T12 and L1 spinal nerves, positioned over the psoas muscle before entering the inguinal region through the transversus abdominis and internal oblique muscles, medial to the anterior superior iliac spine. They then continue their course between the external and internal oblique muscles and their aponeuroses. The ilioinguinal nerve typically runs over the cremaster muscle and exits through the external inguinal ring, whereas the iliohypogastric nerve is positioned more cranially along the internal oblique muscle, eventually reaching the junction with the rectus abdominis fascia.

The genital branch of the genitofemoral nerve, originating from the L1 and L2 spinal nerves, enters the inguinal region via the deep inguinal ring and runs beneath the spermatic cord, covered by the cremasteric fascia, before exiting through the external ring. However, these three nerves are not always present in every individual. A study reported that the ilioinguinal, iliohypogastric, and genital branches of the genitofemoral nerve were identified in 96%, 94%, and 90% of cases, respectively [4].

Significant variations exist in the pathways of these nerves, and their sensory functions frequently overlap. Research on the emergence and distribution of the ilioinguinal nerve identified 16 different branching patterns and 8 distinct distribution types, with a predominant anterior scrotal distribution. This finding suggests that scrotal pain is not solely mediated by the genital branch of the genitofemoral nerve [5].

**Fig. 1:**
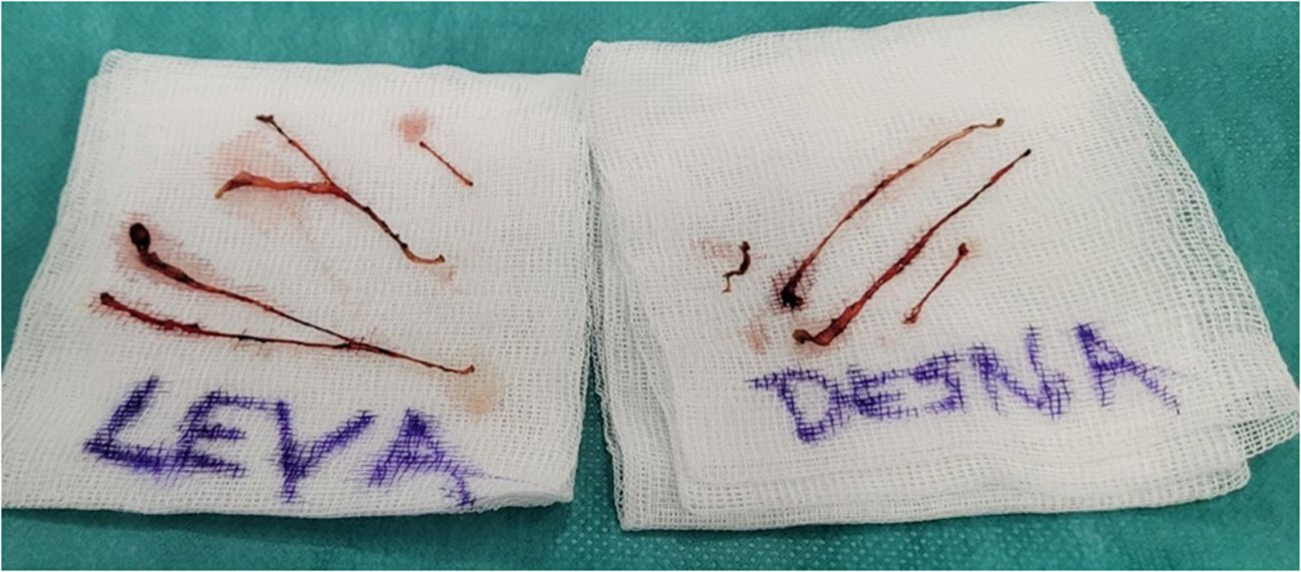
Bilateral triple neurectomy, same patient, variability in nerve branching left vs right

Another study reported that, in addition to cutaneous branches from the ilioinguinal nerve (present in 90.7% of cases), cutaneous branches originating from the genital branch of the genitofemoral nerve were found in the inguinal region in 35.2% of cases. Furthermore, in 13% of cases, the genital branch and the ilioinguinal nerve merged within the inguinal canal [6]. This complex neural anatomy likely explains why patients often describe poorly localized pain, as no single structure appears solely responsible for inguinal-related groin discomfort. This is also the reason why it is difficult to diagnose clinically which nerve is responsible for pain (Fig. 1).

## Aetiology

Various theories have been proposed regarding the underlying causes of inguinal-related groin pain, which can generally be classified into two broad categories: **neuropathic** and **nociceptive**. Neuropathic pain is thought to arise from nerve entrapment due to compression by overlying aponeuroses or bulging of the posterior wall of the inguinal canal. In contrast, nociceptive pain is associated with musculoskeletal causes, such as aponeurotic tears or enthesopathy of the inguinal ligament. These different mechanisms contribute to distinct pain characteristics—neuropathic pain is often described as electric, burning, or tingling, while nociceptive pain tends to present as stabbing, dull, or aching [7, 8].

Although inguinal hernias can sometimes be asymptomatic, in certain cases, they may cause pain due to direct compression of nerves. Histopathological studies have even demonstrated fat degeneration of nerves distal to the site of compression [9]. Research on postoperative pain following inguinal hernia surgery has shown that direct nerve injuries (e.g., transection or suturing) primarily lead to neuropathic pain, whereas postoperative inflammatory responses (such as scar tissue formation or foreign body reactions) contribute to nociceptive pain.

**Fig. 2:**
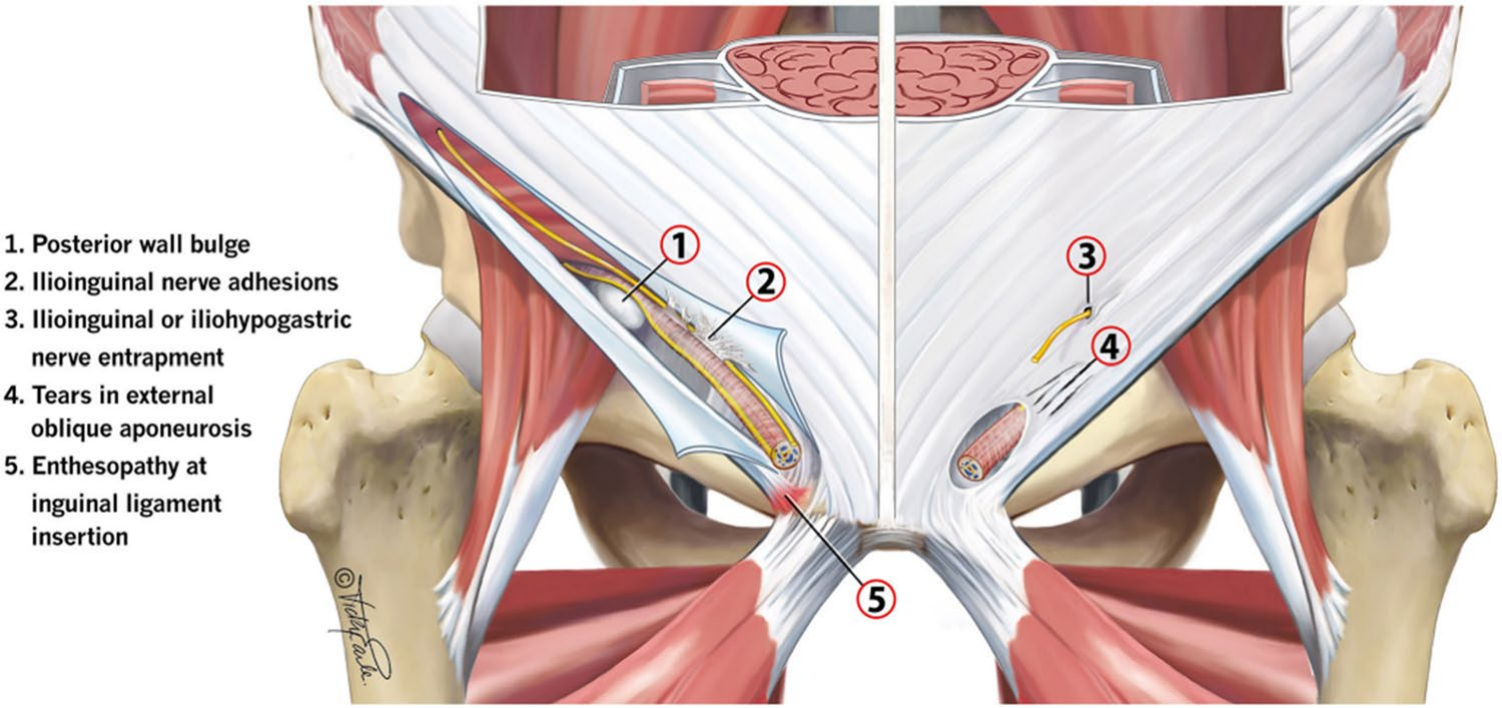
Proposed causes for inguinal-related groin pain (8)

A proposed sequential model of pain development suggests that multiple factors—including genetic predisposition, age, memory, and mental state—play a role in neuroplastic changes following either neuropathic or nociceptive nerve injuries. These factors may explain the variability in pain onset and intensity among individuals who have sustained similar injuries [10]. Importantly, current evidence suggests that the primary source of pain in inguinal-related groin pain is localized within the inguinal canal, where the nerves are located, rather than in the preperitoneal plane. This distinction is crucial when determining the most appropriate treatment approach (Fig. 2).

## Examination

Clinical assessment is essential for diagnosing groin pain, with inguinal-related groin pain being particularly challenging to reproduce during examination. Patients present with diverse histories and symptom patterns. Some athletes experience persistent pain during and after physical activity, typically localized to the inguinal ligament, inguinal posterior wall, oblique muscles, and distal rectus abdominis. This pain distribution aligns with the dermatomes of the ilioinguinal and iliohypogastric nerves and is often described as a dull or stabbing discomfort, consistent with nociceptive pain mechanisms.

Conversely, other patients report minimal discomfort during routine activities but develop sudden, short-lasting pain described as electric or burning sensations when engaging in high-intensity movements such as rapid directional changes, kicking, acceleration, or deceleration. This type of presentation is more indicative of neuropathic pain, likely resulting from direct nerve entrapment or compression. In some cases, the ilioinguinal or iliohypogastric nerves traverse through the external oblique aponeurosis, making them susceptible to entrapment within fibrotic tissue—an adaptation observed more frequently in athletes than in the general population. Additionally, bulging of the posterior inguinal wall is commonly associated with compression of the genital branch of the genitofemoral nerve [10].

Clinical evaluation begins with palpation of the inguinal canal, including invagination of the scrotal skin and digital examination of the inguinal canal while asking the patient to perform a Valsalva manoeuvre. The next step involves muscle resistance tests to assess the strength of the lateral abdominal wall and to determine whether pain can be elicited.

**Fig. 3:**
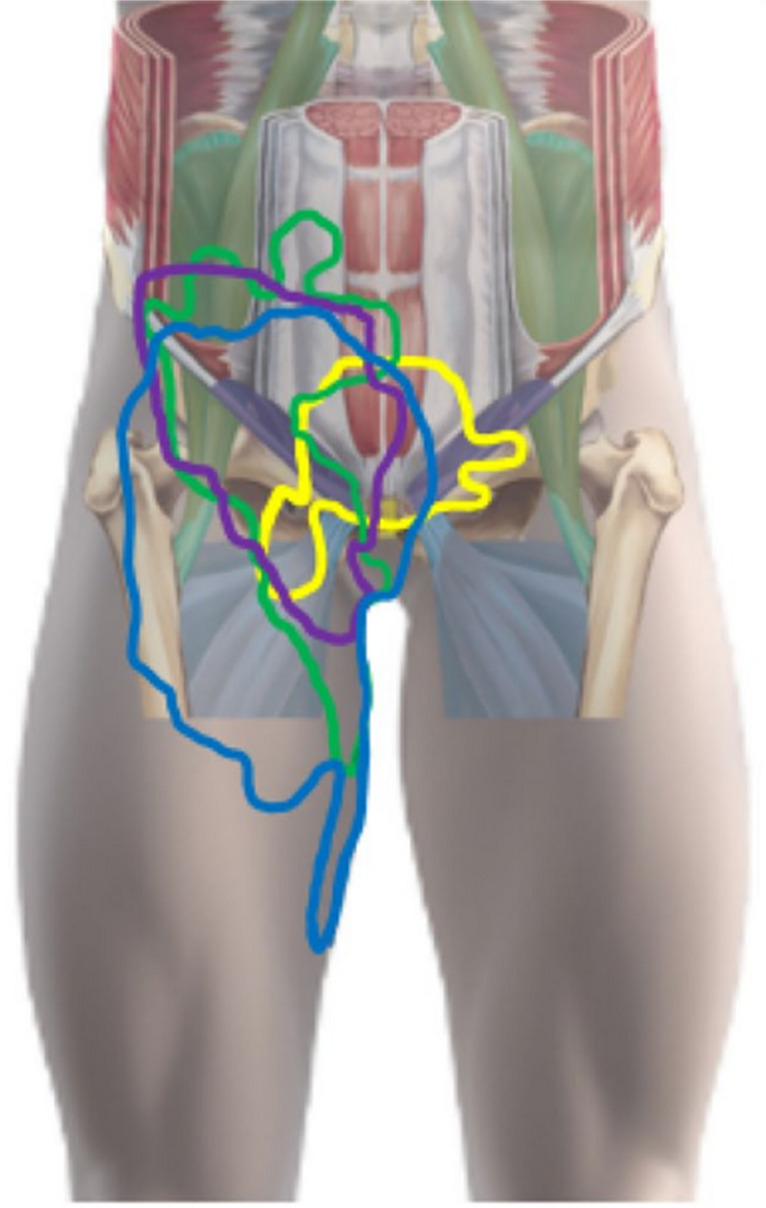
Purple line shows reported inguinal-related groin pain as reported by patients in a study (7)

A recent study investigating the reliability of clinical tests for inguinal-related groin pain found that the Valsalva maneuver was the most commonly positive palpation test, reinforcing its clinical utility. However, no single test was found to be definitively reliable for diagnosing inguinal-related groin pain in athletes [11]. A retrospective study further demonstrated that in 44% of cases, athletes with groin pain exhibited involvement of multiple anatomical structures, underscoring the importance of conducting a comprehensive examination that includes assessment of the adductors, iliopsoas, pubic bones, and hip joints [12] (Fig. 3).

## Imaging

Diagnostic imaging plays a supportive role in evaluating groin pain, as it helps exclude alternative causes and provides insight into the structural integrity of the affected region. However, there is no universally accepted gold standard for imaging in the context of inguinal-related groin pain, which makes interpretation challenging.

The most frequently utilized imaging modalities include X-ray, ultrasound (US), and magnetic resonance imaging (MRI). Each of these techniques offers distinct advantages and limitations.

- X-ray is typically performed initially to rule out bony abnormalities, such as pubic symphysis osteitis, stress fractures, or avulsion injuries. However, X-rays have limited utility in directly visualizing soft tissue pathology within the inguinal region.
- Ultrasound (US) is a dynamic imaging modality that allows for real-time assessment of the inguinal region. It is particularly useful for identifying posterior wall bulging, dynamic inguinal canal changes, and subtle soft tissue abnormalities. However, due to significant operator dependence, ultrasound findings must be interpreted cautiously.
- MRI provides the most comprehensive soft tissue evaluation, enabling visualization of muscle, tendon, and bone marrow changes. Findings such as pubic bone marrow edema and symphysis pubis irregularities are frequently observed in athletes with groin pain. However, these changes are also prevalent in asymptomatic individuals, raising concerns about false positives [13].

A study comparing asymptomatic and symptomatic athletes found that 48% of symptom-free individuals had pubic bone stress changes on MRI, which was nearly identical to the 50% prevalence in non-athletic controls [14]. Similarly, an extensive MRI study of hip and groin pathology in athletes reported that 77% of asymptomatic individuals exhibited positive findings, with specific variations ranging from 36% for adductor-abdominal involvement to 64% for hip-related abnormalities [15].

These findings emphasize the importance of clinical correlation when interpreting imaging results. Simply identifying an abnormality does not confirm it as the source of pain, reinforcing the necessity of a comprehensive clinical assessment alongside imaging studies.

### Treatment

The primary approach to managing inguinal-related groin pain follows a stepwise treatment algorithm, beginning with conservative therapy before progressing to interventional and surgical options when necessary.

### Conservative management

The first-line treatment consists of:

- Activity modification – Temporary cessation of high-intensity sports participation, particularly movements that exacerbate symptoms (e.g., cutting, kicking, sprinting).
- Structured rehabilitation – A supervised, exercise-based program aimed at restoring lateral abdominal wall strength, addressing muscle imbalances, and optimizing core stability. Key components include progressive resistance training, neuromuscular control exercises, and eccentric strengthening of the hip and pelvic stabilizers [16, 17].

Although rehabilitation is often effective, some athletes continue to experience persistent pain, necessitating further interventions.

### Injection therapy

If symptoms persist despite rehabilitation, image-guided nerve blocks targeting the ilioinguinal and iliohypogastric nerves may be considered. These injections typically contain:

- Local anaesthetics for immediate pain relief.
- Corticosteroids to reduce inflammation and stabilize neuronal membranes, thereby prolonging the duration of analgesia.

Nerve blocks have been shown to provide complete symptom resolution in some athletes, while others experience temporary relief lasting one to three months. If pain recurs, the procedure can be repeated at appropriate intervals [18–20].

### Surgical treatment

Surgical intervention is reserved for cases where conservative therapy fails to achieve long-term pain relief. Several operative techniques exist, with approaches differing based on surgeons’ perspectives on the underlying pathology.

Endoscopic (minimally invasive) procedures

Endoscopic repair methods, such as transabdominal preperitoneal (TAPP) and totally extraperitoneal (TEP) repair, involve reinforcing the inguinal canal by placing a mesh within the preperitoneal space.

- A study investigating TEP surgery in athletes with inguinal-related groin pain found that 95% of patients returned to full sports participation within four weeks of the procedure [21].
- A randomized controlled trial (RCT) comparing TEP surgery with conservative treatment demonstrated that 90% of surgically treated athletes achieved pain-free return to sport within three months, whereas only 27% of conservatively managed athletes reached the same outcome [22].

These results indicate that surgery is often more effective than conservative treatment in athletes with persistent symptoms.

Open surgical approaches

Some experts advocate for open surgical techniques, particularly in cases where posterior inguinal wall weakness is suspected.

**Fig. 4:**
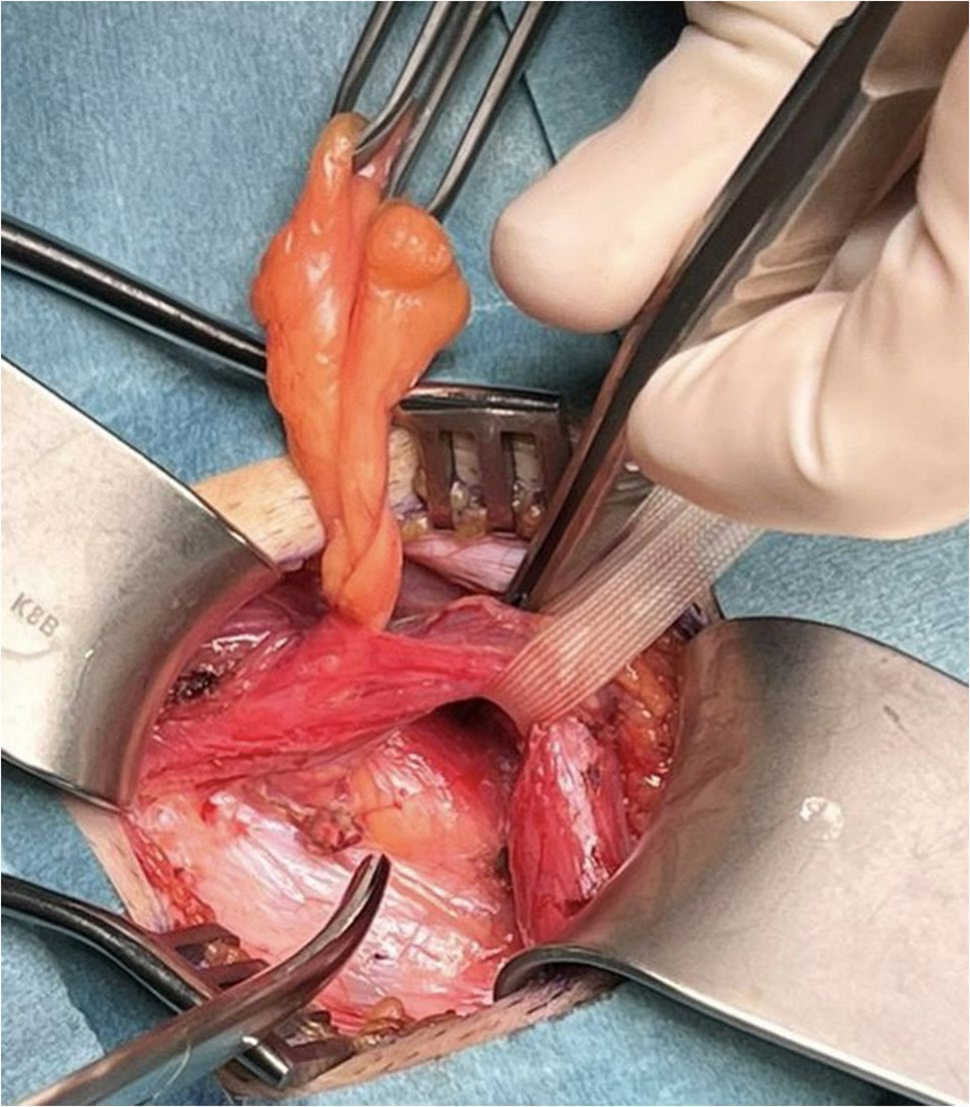
Posterior wall bulge with retroperitoneal lipoma in indirect (lateral) hernia position

- A study comparing Lichtenstein mesh repair with open preperitoneal mesh repair found no significant difference in recovery time, with both groups returning to sports within an average of 53 days postoperatively [23].
- Non-mesh repair techniques, such as the modified Shouldice repair, have also been utilized successfully. One study found that 75% of athletes undergoing open non-mesh repair returned to full activity within 18.5 days, with histopathological analysis confirming perineural fibrosis in all cases [24].
- A recent RCT comparing TEP repair and open suture repair demonstrated similar postoperative pain outcomes and return-to-sport rates, suggesting that both techniques can be effective in treating inguinal-related groin pain [25, 26] (Fig. 4).

### Surgical considerations: mesh vs. non-mesh repair

There is ongoing debate regarding the necessity of mesh reinforcement in athletes with inguinal-related groin pain. Unlike inguinal hernias, where mesh placement is essential for repairing a structural defect, there is no actual hernia in these cases. Therefore, implanting synthetic material may be unnecessary and could increase the risk of fibrosis, scar tissue formation, and nerve entrapment.

Cadaveric studies have highlighted significant variability in nerve trajectories, which may contribute to different clinical presentations. A study analyzing intraoperative findings in athletes undergoing surgery for inguinal-related groin pain reported evidence of nerve compression in the following proportions:

- Ilioinguinal nerve: 96.2%
- Iliohypogastric nerve: 92.5%
- Genital branch of the genitofemoral nerve: 30.8% [10]

**Fig. 5:**
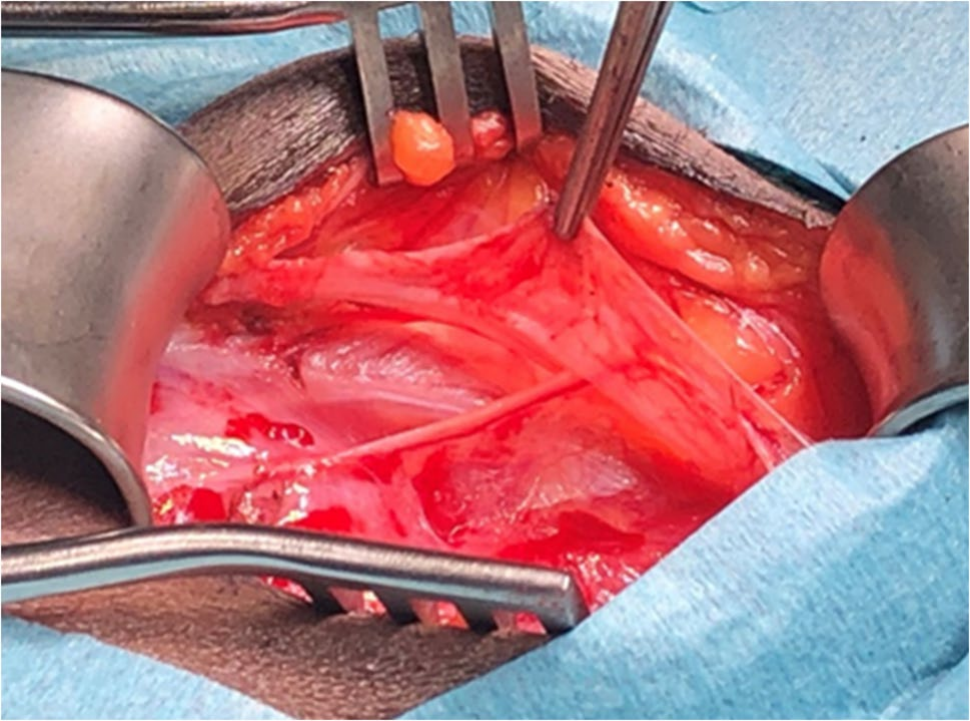
Iliohypogastric nerve entrapment (Right side)

**Fig. 6:**
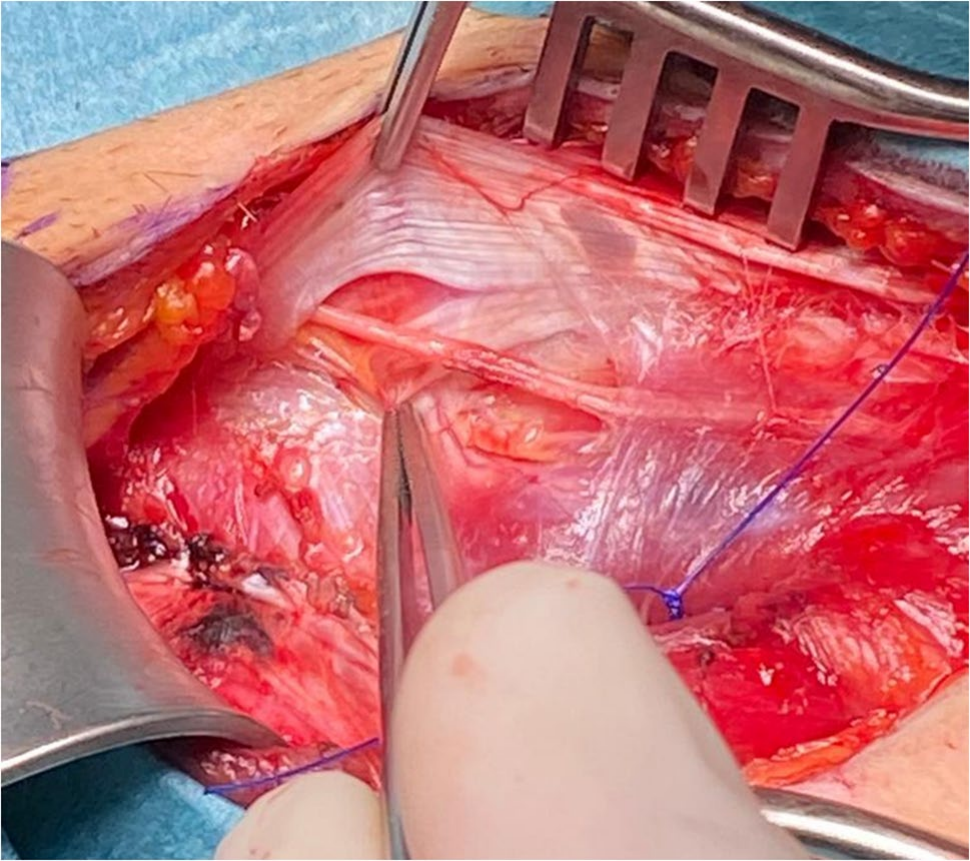
Iliohypogastric nerve entrapment (Left side)

**Fig. 7:**
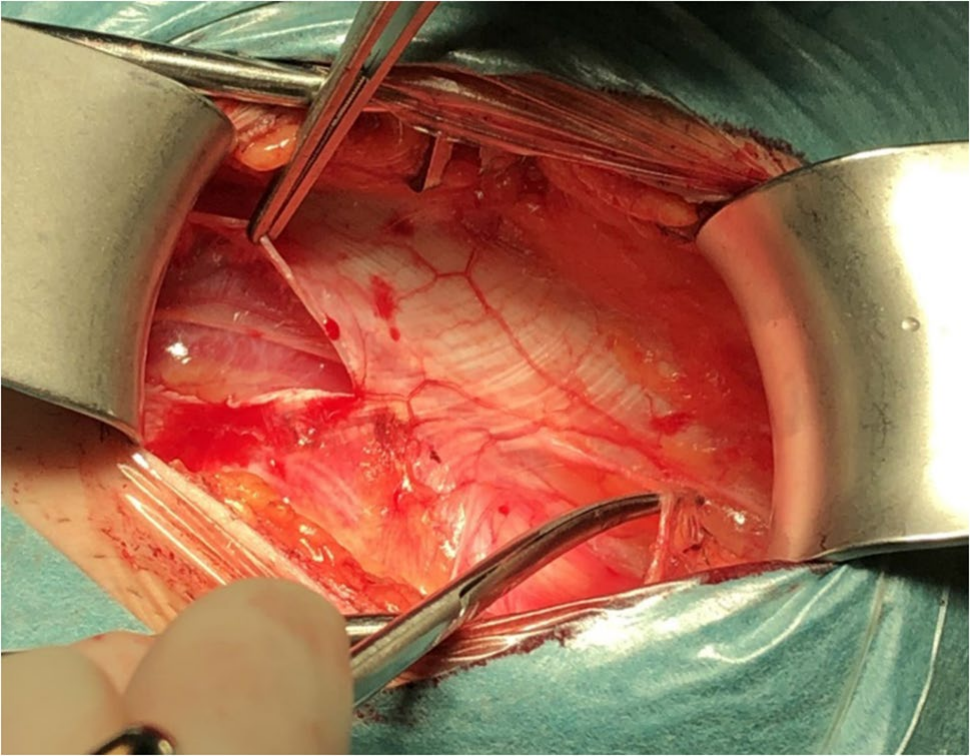
Ilioinguinal nerve entrapment (Right side)

**Fig. 8:**
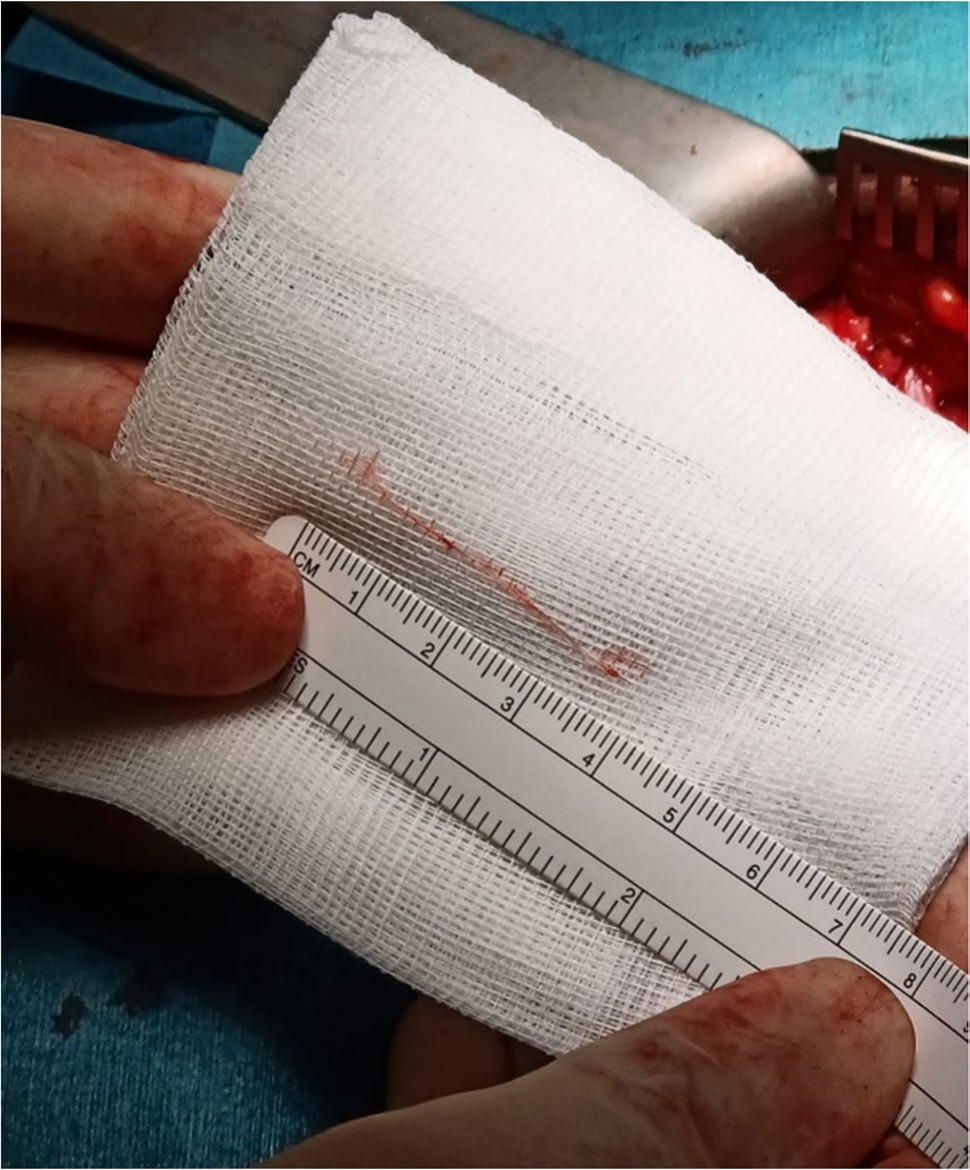
Genital branch of genitofemoral nerve is sometimes difficult to identify

Given this evidence, some surgeons favor open repair with selective neurectomy if necessary, rather than routine mesh placement. Additionally, studies on hernia surgery indicate that identification and preservation of all three inguinal nerves can reduce the risk of chronic postoperative pain. However, general surgeons identify all three nerves in only 40% of cases, whereas experienced hernia specialists report success rates of 70–90% in nerve identification [4] (Figs. 5, 6, 7 and 8).

## Future directions

Further research is required to deepen our understanding of collagen deficiencies [27] in athletes with inguinal-related groin pain, as well as nerve histopathology following neurectomy. Additionally, more randomized controlled trials (RCTs) are needed to compare surgical interventions with and without neurectomies.

There is also a need for studies that directly compare surgical and conservative treatment outcomes, particularly those that clearly define and standardize rehabilitation protocols—an aspect that has often been inadequately described in past research. Furthermore, long-term follow-up studies are essential to obtain a more comprehensive understanding of treatment success rates.

Currently, it remains unclear how many athletes who initially improve with conservative treatment eventually require surgical intervention later in their careers. Similarly, data are lacking regarding the proportion of athletes who undergo surgery for inguinal-related groin pain, return to sport successfully, but then require additional surgical procedures in subsequent years due to recurrent symptoms.

While the precise pathophysiology of inguinal-related groin pain remains partially unresolved, growing evidence suggests that nerve involvement plays a central role. The combination of neuropathic and nociceptive pain characteristics reported by patients further supports this hypothesis.

Future research should focus on improving diagnostic accuracy, optimizing treatment selection, and developing predictive models to identify which athletes are most likely to benefit from conservative therapy versus surgical intervention. By refining our understanding of inguinal-related groin pain, we can enhance clinical decision-making and improve long-term outcomes for affected athletes.

## Data Availability

No datasets were generated or analysed during the current study.
